# Changes in tryptophan breakdown associated with response to multimodal treatment in depression

**DOI:** 10.3389/fpsyt.2024.1380620

**Published:** 2024-06-21

**Authors:** Eva Z. Reininghaus, Melanie Lenger, Elena M. D. Schönthaler, Frederike T. Fellendorf, Tatjana Stross, Markus Schwarz, Natalie Moll, Bernd Reininghaus, Nina Dalkner

**Affiliations:** ^1^ Clinical Division of Psychiatry and Psychotherapeutic Medicine, Medical University of Graz, Graz, Austria; ^2^ Institute of Laboratory Medicine, University Hospital, Ludwig-Maximilian University (LMU), Munich, Munich, Germany

**Keywords:** depression, kynurenine, kynurenine pathway, multimodal treatment, affective disorder

## Abstract

**Background:**

Research on depression showed that dysregulations in tryptophan (TRP), kynurenine (KYN), and its KYN pathway metabolites are key aspects in the development and maintenance of depressive symptoms. In our previous reports, we described sex-specific changes in TRP breakdown as well as changes in KYN and KYN/TRP in association with treatment response and inflammatory and metabolic parameters. However, results of treatment effects on KYN pathway metabolites as well as how pathway changes are related to treatment response remain sparse.

**Objective:**

We investigated potential changes of KYN and KYN pathway metabolites in association with therapeutic response of individuals with depression during a six-week multimodal psychiatric rehabilitation program.

**Methods:**

87 participants were divided into treatment responders and non-responders (48 responders, 39 non-responders; 38 male, 49 female; *M*_age_ = 51.09; *SD*_age_ = 7.70) using scores of psychological questionnaires. KYN pathway metabolites serum concentrations as well as their ratios were collected using high performance liquid chromatography. Changes over time (time of admission (t1) vs. time of discharge (t2)) were calculated using repeated measure analyses of (co)variance.

**Results:**

Non-responders exhibited higher levels of 3-Hydroxyanthralinic acid (3-HAA), nicotinic acid (NA), and 3-HAA/KYN, independently of measurement time. NA levels decreased, while 3-HAA levels increased over time in both groups, independently of treatment response. 3-HK/KYN levels decreased, while KYN levels increased in non-responders, but not in responders over time.

**Discussion:**

The results indicate that some compounds of the KYN pathway metabolites can be altered through multimodal long-term interventions in association with treatment response. Especially the pathway degrading KYN further down to 3-HAA and 3-HK/KYN might be decisive for treatment response in depression.

## 1 Introduction

Depression is a psychiatric disorder, which encompasses symptoms of mood and activity changes (1), and remains one of the main causes of disability worldwide (2). Due to its multifaceted symptomatology, much research has been done to elucidate the underlying causes and identify its biological basis (3). Next to genetic causes, environmental factors (e.g., stressful events), dysregulations in neuroendocrinological systems (e.g., the hypothalamic-pituitary-adrenal axis), or neurotransmitters (e.g., dopamine, noradrenaline), structural and functional brain alterations, and chronic sub-inflammation (4), the metabolism of the amino acid L-tryptophan (TRP) has been shown to play a substantial role in the development of mood disorders (e.g., 5–7).

**Figure 1:**
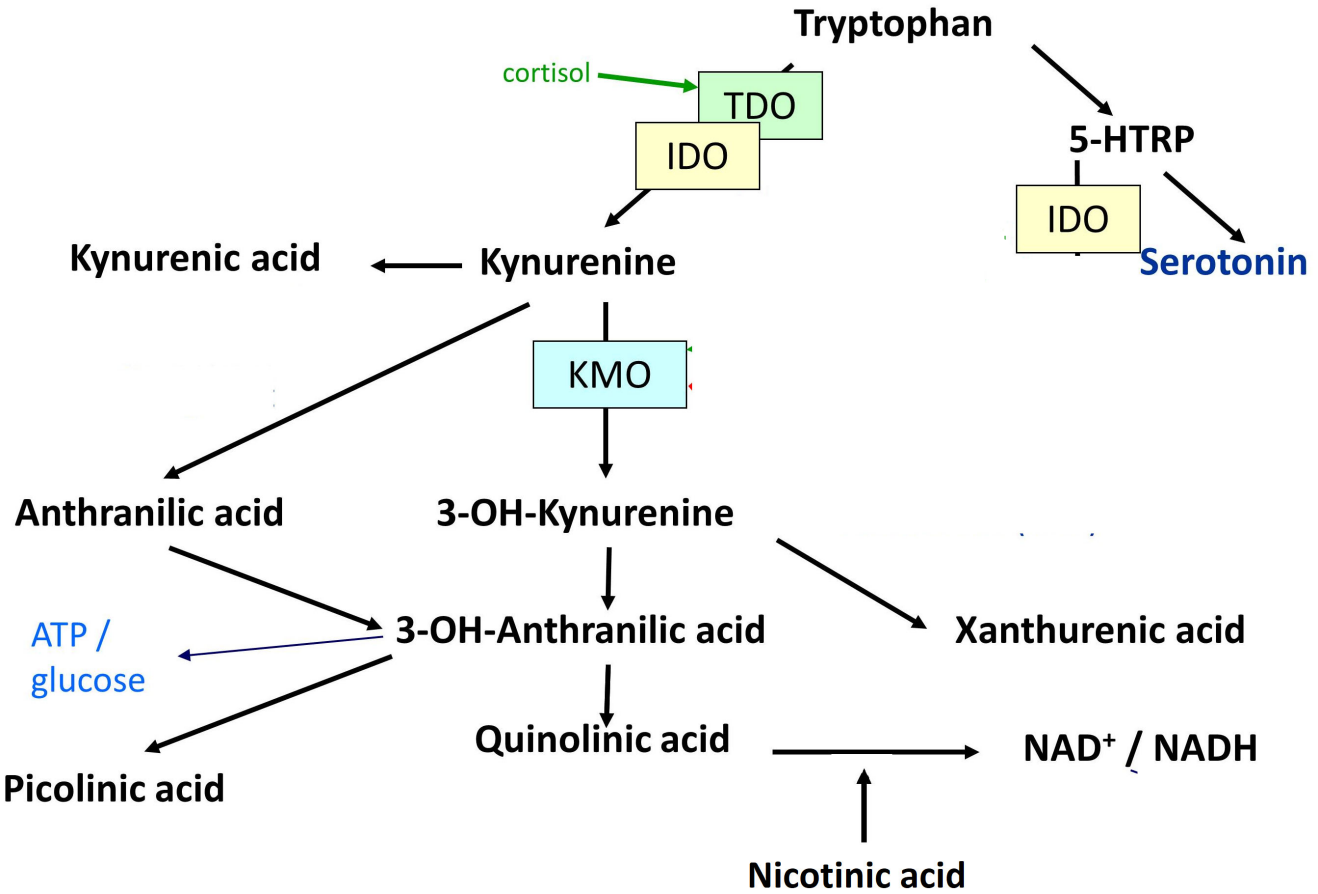
Degradation of tryptophan into kynurenine and serotonin, as well as their downstream metabolites. 5-HTRP = 5-Hydroxytryptophan. TDO = Tryptophan-2,3-dioxygenase. IDO = Indoleamine-2,3-dioxygenase. KMO = Kynurenine-3-monooxygenase. 3-OH-Kynurenine = 3-Hydroxykynurenine. 3-OH-Anthralinic acid = 3-Hydroxyanthralinic acid. NAD+/NADH = Nicotinamide adenine dinucleotide. NMDA-R = N-methyl-D-aspartate agonist. α7nACh-R agonist = Nicotinic acetylcholine agonist. ATP = Adenosine triphosphate. mGlu2/3 agonist = Metabotropic 2/3 agonist.

TRP is best known as a precursor of the neurotransmitter serotonin (5-HT), which has been frequently shown to be disturbed in affective states. In addition, TRP is metabolized through the kynurenine (KYN) pathway (8). An increase in the latter pathway is in many cases associated with and triggered by inflammation (9). The KYN pathway, which metabolizes up to 99% of TRP (10), elicits the production of the KYN metabolites 3-hydroxykynurenine (3-HK), xanthurenic acid (XA), 3-hydroxyanthranilic acid (3-HAA), quinolinic acid (QA), picolinic acid (PA), nicotinic acid (NA), anthralinic acid (AA), and kynurenic acid (KYNA). The pathways of 5-HT, KYN, and their metabolites are referred to as TRP catabolite pathways (11) or KYN pathway metabolites. In microglial cells, KYN is degraded into 3-HK by kynurenine 3-monooxidase (KMO), and further degraded to 3-HAA and QA, which were found to have neurotoxic properties (9, 12). In contrast, kynurenine aminotransferase (KAT) degrades KYN into KYNA in astrocytes, which is putatively neuroprotective. **Figure 1** shows the degradation of TRP into KYN and 5-HT, as well as their downstream metabolites.

The degradation of TRP is furthermore affected by many variables like sex (13, 14), age, weight (15, 16), psychological distress (17), and general health (18). Moreover, certain metabolites in the KYN pathway have neuroprotective effects, which act as antagonists at the n-methyl-d-aspartate (NMDA) receptors, while other metabolites have a neurotoxic impact due to agonistic effects at the NMDA receptors, which induce apoptosis in astrocytes and generate free oxidative radicals (19, 20). Thus, the degradation of TRP has been frequently shown to impact the genesis, symptomatology, and illness course in neuropsychiatric disorders (21, 22). While a higher prevalence of neurotoxic KYN pathway metabolites was shown to lead to deteriorated cognitive performance (23, 24), poorer psychiatric medication response (20), and more somatic comorbidities (25, 26), a higher concentration of neuroprotective KYN pathway metabolites was reported to possess anti-depressant effects (27).

In the context of depression, chronic low-grade inflammation is known as an important factor in the pathophysiology (reviewed in 28). Thus, the KYN pathway deserves particular attention in the field of affective disorders. The role of such inflammatory processes has already long ago been shown in the emergence of depression in interferon-α-treated hepatitis C patients (29–32). Decreased levels of central 5-HT, which have classically been associated with mood disorders, may therefore be driven by systemic inflammatory processes (9, 33). These findings are corroborated by a large body of studies, which found alterations in TRP, 5-HT, KYN, and KYN pathway metabolites in individuals with depression. For instance, a meta-analysis of 101 studies reported that TRP, KYN, the KYNA/QA ratio, and the KYNA/3-HK ratio were decreased in depression, suggesting a shift in the TRP metabolism from the 5-HT to the KYN pathway (34). Specifically, it was shown that neurotoxic KYN pathway metabolites were related to more severe depressive symptoms (35–37), while neuroprotective KYN pathway metabolites like KYNA and the KYNA/KYN ratio were decreased in individuals with depression (38). This indicates that not only the reduced breakdown from TRP into 5-HT and KYN, but also the concentration of KYN pathway metabolites and the relative ratio of these products, which reflects the neuroprotective/neurotoxic potential (39), might play an important role in the pathophysiology of depression. Finally, overproduced proinflammatory cytokines in depression induce the indoleamine 2-3-dioxygenase (IDO) enzyme, which promotes the KYN pathway, and decreases the activation of the 5-HT pathway. Thus, the KYN pathway is an important therapeutic target in depression (5).

Although many studies suggest changes in the KYN pathway after pharmacological treatment, there is only a small number reporting on the changeability of the KYN pathway before and after multimodal treatment. In one of our previous studies, we found sex-specific changes in the TRP breakdown during psychiatric multimodal treatment over a six-week period (7). There was a significant difference between women and men regarding the changes in TRP, KYN, and KYN/TRP over time, even if controlled for relevant covariates. Particularly, men showed a significant increase in KYN and KYN/TRP compounds over time. In another study, we were able to find changes in KYN/TRP in association to treatment response (40). Specifically, KYN was found to increase in individuals, who did not respond to treatment, while the KYN/TRP ratio decreased over time in individuals responding to treatment. Furthermore, changes in KYN as well as high sensitivity C-reactive protein (hsCRP) levels correlated significantly with changes in the body mass index (BMI) over time, which underlines the involvement of inflammatory processes.

In the present study we aimed to provide a deeper look into the potential changes of KYN pathway metabolites in association with therapeutic response of individuals with depression. After showing changes of the KYN/TRP ratio depending on treatment response (40), we now focused on changes of the detrimental KYN pathway metabolites further down in the KYN pathway over the course of a six-week rehabilitation program in individuals with life-time major depressive disorder and current depressive symptomatology. Notably, the purpose of this study was to investigate the correlative, not the causal relationship between treatment response and KYN pathway metabolites. For this purpose, KYN, KYNA, 3-HK, XA, 3-HAA, QA, PA, NA, AA as well as their ratios were analyzed at the time of treatment admission (t1) and discharge (t2). It was hypothesized that there are significant differences between individuals responding to treatment (responders) and individuals not responding to treatment (non-responders) in KYN pathway metabolite levels over time.

## 2 Methods

### 2.1 Sample

The present study was conducted at an Austrian psychiatric rehabilitation center with treatment focus on affective and stress-related disorders, between April 2015 and April 2017. Since this study was part of a larger project assessing psychiatric symptoms, anthropometric measures, blood samples, psychological testing, and lifestyle questionnaires in this rehabilitation setting, data of 600 individuals were originally available. Exclusion criteria were a main diagnosis of schizophrenia, neurodegenerative disorders, and substance disorders. Participants were included if they were of legal age, had a life-time history of a unipolar affective disorder (F32 and F33 according to ICD-10 diagnosis; 1) and current moderate to severe depressive symptoms according to clinical evaluations and psychological questionnaires (see 2.2. Materials). In a second step, extreme groups (i.e., responders and non-responders) were filtered out according to changes in depression scores (see 2.3. Procedure). In total, 87 individuals (38 male, 49 female; *M*_age_ = 51.09; *SD*_age_ = 7.70) were included into the data analyses. A *post-hoc* power analysis (G*Power 3.1.9.4; 41) showed that for this sample size, an effect size of *f* ^2 ^= 0.3, an α-level of 5%, and two groups with two measurements within a repeated univariate analysis of co-variance (ANCOVA), the achieved power level is .99. The study was approved by the local ethics committee (Medical University of Linz, Upper Austria) and was conducted in compliance with the Declaration of Helsinki and ICH guideline for Good Clinical Practice (EC-number: E-24–14). All participants were informed about the study at the time of admission and gave written informed consent prior to their participation in the study. The study used the sample of the already published paper on changes in TRP and KYN in association to treatment response (40), and additionally analyzed KYN pathway metabolites.

### 2.2 Materials

#### 2.2.1 Beck depression inventory – II 

Current depressive symptom severity was assessed with the self-report questionnaire BDI-II, which comprises 21 items representing single depressive symptoms (42). Participants were asked to rate their perceived depression severity on a four-point Likert scale, ranging from 0 to 3 at the time of admission as well as discharge. A sum score was built to indicate depression severity, with higher scores indicating greater depression severity (minimum score = 0, maximum score = 63).

#### 2.2.2 Biological assays/quantification of KYN pathway metabolites

To measure KYN and KYN downstream metabolites, fasting blood samples were taken between 8.00 and 10.00 am. Blood samples were either processed immediately for further analysis or stored at -80° C until thawed for biological assays. All analyses were performed under the identical chromatographic conditions and standards (see 43).

Sample preparation: Aliquots of 120 µl of serum samples, calibrators and quality controls were used for sample preparation each. After adding 40 µl of the internal standard (ISTD) mixture, protein precipitation was carried out in two steps by subsequently adding 150 µl methanol/ethanol (2/1 v:v) and 400 µl acetonitrile. After centrifugation, the supernatants were evaporated to dryness and reconstituted in mobile phase A.

LC-MS/MS conditions: The chromatographic system consisted of a Waters Acquity UPLC separations module connected to a Xevo TQ MS triple-quadrupole mass spectrometer (Waters Corp., Milford, MA, USA). Separation was carried out using a Kinetex XB-C18, 2.6 µm, 2.1 x 150 mm column (Phenomenex, Torrance, CA, USA). System operation, data acquisition, and data processing were carried out using MassLynx V4.1 software (Waters Corp.).

Mobile phase A was composed of 0.1% formic acid and 0.01% HFBA in water, mobile phase B was methanol. Flow rate was set at 0.25 ml/min, column temperature was set at 30.0°C. For chromatography, 7.5 µl of the reconstituted samples, calibrators, and controls were loaded onto the LC-MS/MS system.

The Xevo TQ MS was operated in atmospheric pressure and in positive (ESI+) electrospray ionization mode. The analytes and ISTDs were detected using multiple reaction monitoring (MRM) and scanned in small retention time windows to optimise scanning quality.

The Lower Limit of Quantification and Lower Limit of Detection of the used method were calculated according to DIN 32645 guidelines. The method was further validated based on the EMEA guidelines at the Institute of Laboratory Medicine, Medical Center of Ludwig Maximilian University, Munich, Germany.

### 2.3 Procedure

The study design included the comprehensive evaluation of depressive symptoms (BDI-II), KYN, and KYN downstream metabolites concentrations, as well as their ratios at admission (t1) and discharge (t2) of a six-week rehabilitation program. Data of individuals fulfilling the inclusion criteria were selected by an extreme group comparison. Participants exhibiting a drastic reduction in BDI-II scores from severe to non/minimal depressive symptoms (BDI-II > 29 to BDI-II < 14) within their rehabilitation stay were classified as responders (*n* = 48), while those with minimal or no changes in their BDI-II score (BDI-II ≥ 20 and less than a four-point difference from t1 to t2) were classified as non-responders (*n* = 39). In between measurements (t1 and t2), all participants completed the rehabilitation therapy program including weekly medical consultations, psychotherapy, occupational therapy, physical training, and diet counseling. Therapy was either performed in a single or group setting with up to 18h – 20h of therapy weekly. In general, all patients received a similarly structured and targeted rehabilitation treatment.

### 2.4 Statistical analysis

Data were analyzed using the statistical software SPSS (Version 29). First, group differences in demographic variables, medical data (smoking severity, Body-Mass-Index (BMI)), psychological data (BDI-II scores), KYN, and KYN downstream metabolites as well as the according ratios between responders and non-responders were calculated using χ^2^-tests and *t*-tests. Secondly, to test the main hypotheses, univariate repeated measure analysis of (co)-variance (RM-AN(C)OVA) were performed using the between-factor group (responder versus non-responder), the within-factor time (t1 versus t2), the dependent variables KYN, the KYN downstream metabolites, and their ratios. Differences in BMI (BMI_diff_) between t1 and t2 were previously found to be associated with KYN (see 40) and were thus included as a covariate in the RM-ANOVA analysis regarding KYN to account for potentially confounding effects. Assumptions for conducting all analyses were fulfilled or the analyses methods were corrected accordingly. Regarding the assumption of normality, non-normal data were not transformed, since the robustness of ANOVAs against non-normality was previously shown to be given, if variance homogeneity assumptions are met (44). Moreover, outliers were not dropped from the analyses unless they appeared as impossible values, since they represent naturalistic observations. However, violations of homoscedasticity were corrected using adequate transformations. All analyses were tested at a significance level of α = .05. Data and data codes can be accessed via https://doi.org/10.17605/OSF.IO/JDHY5.

## 3 Results

### 3.1 Descriptive analyses

Results of the descriptive analyses showed no significant differences in age, sex, BMI (at t1), and smoking severity between responders and non-responders at the time of admission (see **Table 1**). The severity of depressive symptoms (BDI-II score) was significantly higher in the responder group than in the non-responder group at t1, as already described in Reininghaus et al. (40).

**Table 1:** Descriptive statistics of demographic, medical, and psychological data at the time of admission (t1).

Parameter (*M, SD*)	Non-Responder (*n*=39)	Responder (*n*=48)	Statistics
Age	51.31 (8.40)	50.90 (7.18)	*T*(85) = 0.24, *p* = .808
Females in % (*n*)	56.40 (22)	56.30 (27)	χ^2^(1) = 0.00, *p* = .988
BMI [kg/m^2^]	27.57 (4.61)	26.48 (4.52)	*T*(83) = 1.22, *p* = .227
Smoking severity	0.79 (1.88)	1.00 (1.83)	*T*(84) = − 0.51, *p* = .611
Tryptophan[ng/ml]	12550.94 (2067.72)	12966.53 (1829.46)	*T*(81) = - 0.97, *p* = .335
hsCRP [mg/l]	2.44 (2.56)	1.76 (1.74)	*T*(64.41) = 1.39*, p* = .169
BDI-II	27.41 (5.51)	33.41 (4.42)	*T*(85) = - 5.96**, *p* < .01

M, Mean; SD, Standard deviation; BMI, Body Mass Index; Smoking severity, Measured by the Fagerstroem Test; hsCRP, High-sensitivity C-reactive protein; BDI-II, Beck Depression Inventory-II Score; Significant results are printed in bold. **p < .01.

Regarding the descriptive analyses of KYN, KYN downstream metabolites, and their ratios, significant differences were found for 3-HAA and 3-HK/KYN ratios between responders and non-responders at the time of admission (see **Table 2**). Specifically, 3-HAA concentrations were significantly lower in the responder group than in the non-responder group. Moreover, non-responders exhibited higher ratios of 3-HAA/KYN and 3-HK/KYN than responders. For the time of discharge, significant differences between responders and non-responders were found for 3-HAA, AA, and AA/KYN ratios (see **Table 3**). Specifically, non-responders had higher levels of 3-HAA, AA, and AA/KYN ratios than responders. No significant group differences were observed for the other compounds of the KYN pathway.

**Table 2:** Descriptive statistics of kynurenine, kynurenine downstream metabolites, and their ratios at admission (t1).

	Non-Responder *M* (*SD*)	Responder *M* (*SD*)	Statistics
KYN [ng/ml]	423.81 (103.34)	445.91 (119.33)	*T*(81) = -0.87, *p* = .378
3-HK [ng/ml]	17.02 (5.35)	15.62 (4.67)	*T*(81) = 1.28, *p* = .206
3-HAA [ng/ml]	9.45 (3.22)	7.93 (1.81)	*T*(44.4) = 2.41*, *p* = .010
QA [ng/ml]	64.14 (21.39)	66.13 (32.08)	*T*(81) = -0.32, *p* = .218
NA [ng/ml]	16.25 (8.22)	13.30 (8.41)	*T*(80) = 1.58, *p* = .116
KYNA [ng/ml]	7.88 (3.02)	8.20 (2.97)	*T*(81) = -0.49, *p* = .624
AA [ng/ml]	2.43 (1.00)	2.31 (0.68)	*T*(51.48) = 0.57, *p* = .569
XA [ng/ml]	2.61 (1.98)	2.37 (1.17)	*T*(79) = 0.69, *p* = .501
PA [ng/ml]	19.54 (10.27)	20.43 (11.98)	*T*(81) = -0.36, *p* = .723
3-HK/KYN	0.041 (0.01)	0.036 (0.01)	*T*(81) = 2.38**, *p* = .020
3-HAA/KYN	0.0190 (0.01)	0.0189 (0.01)	*T*(77) = 2.06*, *p* = .043
KYNA/KYN	0.0190 (0.01)	0.0189 (0.01)	*T*(81) = -0.06, *p* = .950
3-HK/KYNA	2.32 (0.78)	2.04 (0.68)	*T*(81) = 1.75, *p* = .084
KYNA/QA	0.13 (0.05)	0.14 (0.09)	*T*(81) = -0.80, *p* = .285
AA/KYN	0.34 (0.20)	0.31 (0.14)	*T*(72) = 0.86, *p* = .395

KYN, Kynurenine; XA, Xanthurenic Acid; KYNA, Kynurenic acid; 3-HAA, 3-Hydroxyanthranilic acid; NA, Nicotinic acid; PA, Picolinic acid; QA, Quinolinic acid; AA, Anthralinic acid; 3-HK, 3-Hydroxykynurenine; 3-HK/KYN ratio, 3-Hydroxykynurenine/kynurenine; 3-HAA/ KYN ratio, 3-Hydroxyanthranilic acid/kynurenine; KYNA/ KYN ratio, Kynurenic acid/kynurenine; 3-HK/KYNA ratio, 3-Hydroxykynurenine/kynurenic acid; KYNA/QA ratio, Kynurenine/quinolinic acid; AA/KYN, Anthralinic acid/kynurenine. Significant results are printed in bold. *p < .05, **p < .01.

**Table 3:** Descriptive statistics of kynurenine, kynurenine downstream metabolites, and their ratios at discharge (t2).

	Non-Responder *M* (*SD*)	Responder *M* (*SD*)	Statistics
KYN [ng/ml]	458.92 (125.67)	430.94 (95.63)	*T*(82) = 1.16, *p* = .250
3-HK [ng/ml]	16.86 (5.04)	15.83 (6.65)	*T*(82) = 0.79, *p* = .435
3-HAA [ng/ml]	10.35 (4.10)	8.01 (2.34)	*T*(46.97) = 2.99**, *p* = .004
QA [ng/ml]	63.95 (20.99)	58.72 (18.01)	*T*(82) = 1.23, *p* = .224
NA [ng/ml]	14.36 (9.92)	10.16 (8.98)	*T*(79) = 1.99, *p* = .050
KYNA [ng/ml]	7.93 (2.74)	7.92 (2.60)	*T*(82) = 0.10, *p* = .992
AA [ng/ml]	2.61 (1.22)	2.01 (0.52)	*T*(39.51) = 2.61**, *p* = .013
XA [ng/ml]	2.81 (2.07)	2.19 (1.20)	*T*(81) = 0.87, *p* = .387
PA [ng/ml]	19.74 (8.52)	20.13 (10.78)	*T*(82) = -0.18, *p* = .855
3-HK/KYN	0.0374 (0.01)	0.0370 (0.01)	*T*(82) = 0.15, *p* = .878
3-HAA/KYN	0.023 (0.01)	0.019 (0.01)	*T*(77) = 1.85, *p* = .069
KYNA/KYN	0.0176 (0.01)	0.0186 (0.01)	*T*(82) = -0.91, *p* = .368
3-HK/KYNA	2.22 (0.58)	2.19 (1.39)	*T*(82) = 0.12, *p* = .902
KYNA/QA	0.14 (0.05)	0.14 (0.07)	*T*(82) = -1.07, *p* = .286
AA/KYN	0.35 (0.16)	0.28 (0.11)	*T*(52.49) = 2.08*, *p* = .043

KYN, Kynurenine; XA, Xanthurenic Acid; KYNA, Kynurenic acid; 3-HAA, 3-Hydroxyanthranilic acid; NA, Nicotinic acid; PA, Picolinic acid; QA, Quinolinic acid; AA, Anthralinic acid; 3-HK, 3-Hydroxykynurenine; 3-HK/KYN ratio, 3-Hydroxykynurenine/kynurenine; 3-HAA/ KYN ratio, 3-Hydroxyanthranilic acid/kynurenine; KYNA/ KYN ratio, Kynurenic acid/kynurenine; 3-HK/KYNA ratio, 3-Hydroxykynurenine/kynurenic acid; KYNA/QA ratio, Kynurenine/quinolinic acid; AA/KYN, Anthralinic acid/kynurenine. Significant results are printed in bold. *p < .05, **p < .01.

### 3.2 Group differences in KYN downstream metabolite levels between measurement times

**Figure 2:**
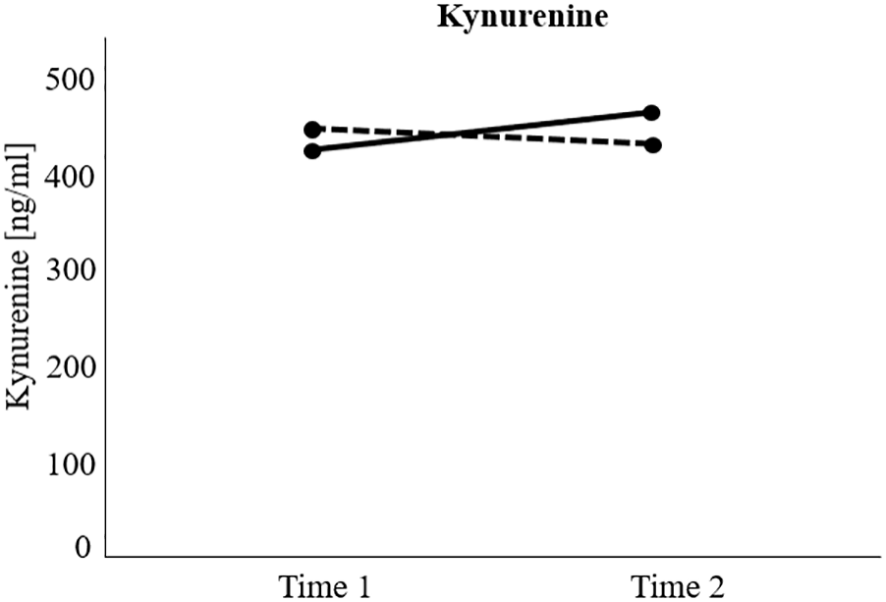
Visualization of significant interaction effect of the repeated measure analyses of (co)variance investigating treatment response (responders versus non-responders) and time differences (measurement time 1 versus measurement time 2) regarding kynurenine levels over time. Non-responders = Black solid line. Responders = Black dotted line. Kynurenine increased significantly over time in the non-responder group, but not in the responder group.

The RM-ANCOVA examining changes in KYN over time between responders and non-responders showed a significant interaction effect group x time (*F*(1, 80) = 7.12, *p* <.01), but no significant main effects of group (*F*(1, 80) = 0.06, *p* = .804), or time (*F*(1, 80) = 1.49, *p* = .225). Specifically, *post-hoc* Bonferroni-corrected pairwise comparisons showed a significant increase in KYN in the non-responder group over time (*M*_(t1)_ = 423.93, *SD*_(t1)_ = 18.88; *M*_(t2)_ = 463.98, *SD*_(t2)_ = 18.21, *p* <.01), but not in the responder group (*M*_(t1)_ = 445.82, *SD*_(t1)_ = 16.53; *M*_(t2)_ = 430.86, *SD*_(t2)_ = 15.94, *p* = .258). For a visualization of the effect, see **Figure 2**.

After using the logarithmic transformation due to violation of the homogeneity assumption, results on 3-HAA indicated a significant main effect of group (*F*(1, 74) = 10.63, *p* <.01), and time (*F*(1, 74) = 4.58, *p* <.05), but no significant interaction effect group x time (*F*(1, 74) = 1.57, *p* = .214). Independently of measurement time, non-responders exhibited higher levels of 3-HAA (*M* = 0.98, *SD* = 0.02) than responders (*M* = 0.90, *SD* = 0.02, *p* <.01). Moreover, 3-HAA significantly increased in both groups over time (*M*_(t1)_ = 0.93, *SD*_(t1)_ = 0.01; *M*_(t2)_ = 0.95, *SD*_(t2)_ = 0.02, *p* <.05).

Regarding NA, the RM-ANOVA showed a significant main effect of group (*F*(1, 77) = 4.74, *p* <.05), and time (*F*(1, 77) = 4.80, *p* <.05), but no significant interaction effect group x time (*F*(1, 77) = 0.22, *p* = .643). Particularly, *post-hoc* pairwise comparisons indicated significantly higher levels of NA in the non-responder group (*M* = 15.39, *SD* = 1.30) than in the responder group (*M* = 11.73, *SD* = 1.07, *p* <.05). In general, NA levels significantly decreased over time in both groups (*M*_(t1)_ = 14.86, *SD*_(t1)_ = 0.97; *M*_(t2)_ = 12.26, *SD*_(t2)_ = 1.08, *p* <.05).

Further, a RM-ANOVA showed a significant interaction effect group x time regarding 3-HK/KYN (*F*(1, 81) = 5.38, *p* <.05), but no significant main effects of group (*F*(1, 81) = 1.37, *p* = .246), or time (*F*(1, 81) = 1.64, *p* = .204). *Post-hoc* pairwise comparisons indicated a significant decrease in the 3-HK/KYN ratio over time in non-responders (*M*_(t1)_ = 0.041, *SD*_(t1)_ = 0.00; *M*_(t2)_ = 0.037, *SD*_(t2)_ = 0.0, *p* <.05), but no significant change in responders (*M*_(t1)_ = 0.04, *SD*_(t1)_ = 0.00; *M*_(t2)_ = 0.04, *SD*_(t2)_ = 0.00, *p* = .433). Since the assumption of variance-covariance homogeneity was not fulfilled in this analyses (Box’ *M* = 17.21, *p* <.001), the more conservative Pillai’s trace was used to estimate the *F*-statistics (45).

**Figure 3:**
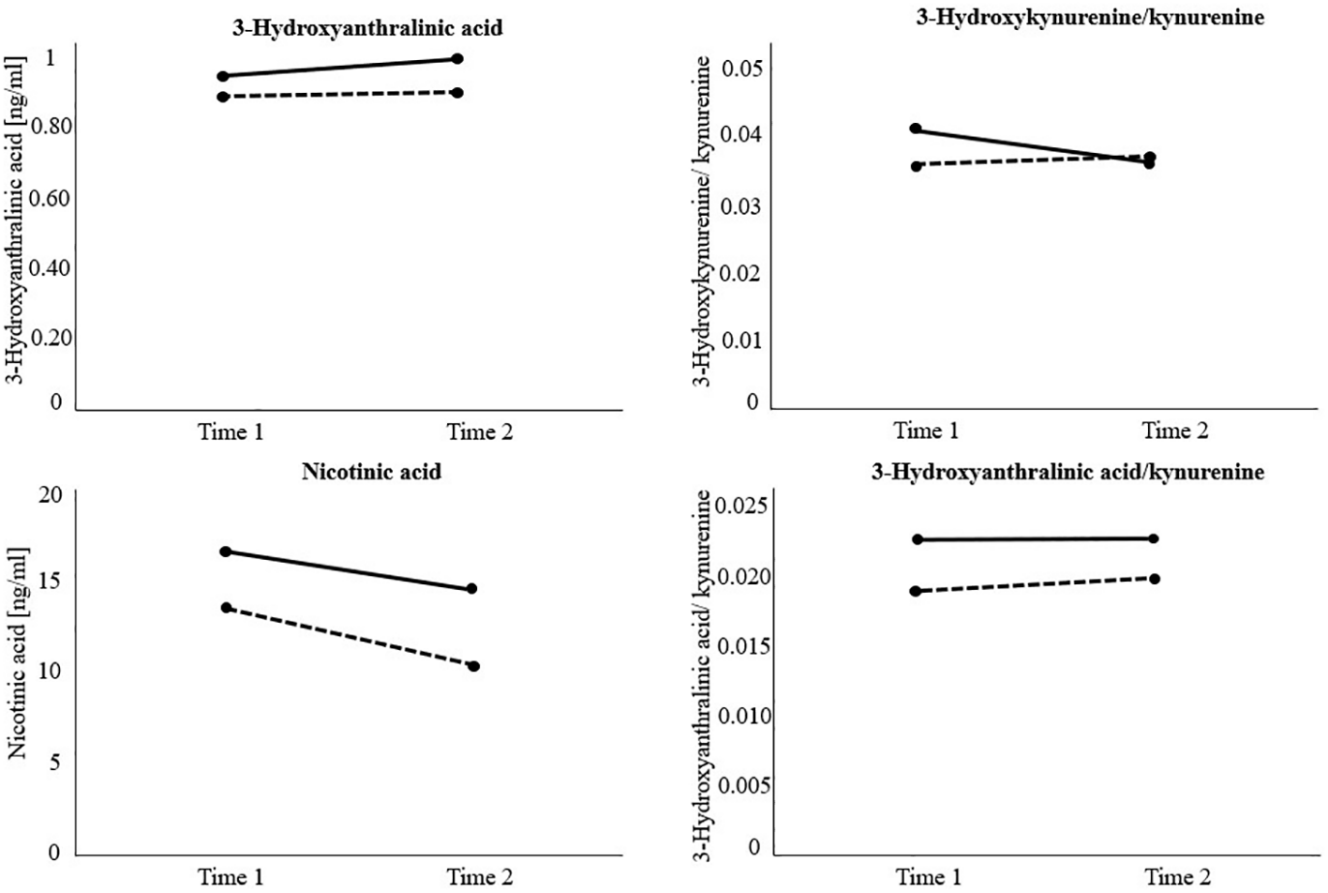
Visualization of significant main and interaction effects of the repeated measure analyses of (co)variance investigating treatment response (responders versus non-responders) and time differences (measurement time 1 versus measurement time 2) regarding kynurenine (KYN) downstream metabolite levels and their ratios over time. Non-responders = Black solid line. Responders = Black dotted line. The variable 3-Hydroxyanthralinic acid (3-HAA) was previously transformed using the logarithmic transformation due to violation of homogeneity of variance. Results indicate significantly higher 3-HAA, nicotinic acid (NA), and 3-HAA/KYN levels in non-responders at each measurement time. Moreover, 3-HAA significantly increased and NA levels significantly decreased in both groups over time. Hydroxykynurenine(3-HK)/KYN significantly decreased in non-responders over time.

Finally, the RM-ANOVA calculating the group difference in 3-HAA/KYN ratios over time indicated a significant main effect of group (*F*(1, 74) = 4.70, *p* <.05), but no significant main effect of time (*F*(1, 74) = 0.48, *p* = .493), or interaction effect of group x time (*F*(1,74) = 0.15, *p* = .696). More specifically, non-responders (*M* = 0.023, *SD* = 0.00) exhibited significantly higher 3-HAA/KYN ratios than responders (*M* = 0.019, *SD* = 0.00, *p* <.05), independently of measurement time. All other analyses did not reach statistical significance. Detailed results can be found in **Table 4**. Visualizations of significant main and interaction effects are displayed in **Figure 3**.

**Table 4:** Results of the repeated measure analyses of (co)variance investigating group (responder versus non-responder) and time differences (measurement time 1 versus measurement time 2) regarding kynurenine, its downstream metabolite levels, and their ratios over time.

	Main effect group		Main effect time		Interaction effect group x time		
	*F*	*η²*	*F*	*η²*	*F*	*η²*	*df*
KYN^a^	0.06	.00	1.49	.02	7.62*	**.09**	1, 80
XA	2.46	.03	.01	.00	1.48	.02	1, 75
KYNA	0.74	.01	0.17	.00	0.74	.01	1, 81
3-HAA^b^	10.63**	**.13**	4.58*	**.06**	1.57	.02	1, 74
NA	4.74*	**.06**	4.80*	**.06**	0.22	.00	1, 77
PA	0.05	.00	0.03	.00	0.31	.00	1, 81
QA	0.15	.00	2.84	.03	3.46	.04	1, 81
AA^c^	0.94	.01	1.48	.02	1.86	.03	1, 70
3-HK	1.22	.02	0.00	.00	0.14	.00	1, 81
3-HK/KYN	1.37	0.02	1.64	.02	5.38*	**.06**	1, 81
3-HK/KYNA	0.64	.01	0.15	.00	1.72	.02	1, 81
KYNA/KYN	0.18	.00	2.29	.03	1.15	.01	1, 81
KYNA/QA	0.93	.01	0.07	.00	0.01	.00	1, 81
3-HAA/KYN	4.70*	**.06**	0.48	.01	0.15	.00	1, 74
AA/KYN ^c^	1.01	.01	1.91	.02	1.34	.00	1, 70

KYN, Kynurenine; XA, Xanthurenic Acid; KYNA, Kynurenic acid; 3-HAA, 3-Hydroxyanthranilic acid; NA, Nicotinic acid; PA, Picolinic acid; QA, Quinolinic acid; AA, Anthralinic acid; 3-HK, 3-Hydroxykynurenine; 3-HK/KYN ratio, 3-Hydroxykynurenine/kynurenine; 3-HK/KYNA ratio, 3-Hydroxykynurenine/kynurenic acid; KYNA/ KYN ratio, Kynurenic acid/kynurenine; KYNA/QA ratio, Kynurenine/quinolinic acid; 3-HAA/KYN ratio, 3-Hydroxyanthranilic acid/kynurenine; AA/KYN, Anthralinic acid/kynurenine. Significant results are printed in bold. *p < .05 **p < .01. ^a^Analysis included the covariate Body Mass Index. ^b^Variable transformed using the logarithmic transformation due to violation of homogeneity of variance. ^c^Variable transformed using the reciprocal transformation due to violation of homogeneity of variance.

## 4 Discussion

The aim of this study was to investigate changes in the KYN downstream metabolites pathways in individuals with depression undergoing multimodal treatment comparing individuals responding and not responding to treatment. For this purpose, KYN, KYN downstream metabolites, and their ratios were analyzed before and after a six-week rehabilitation stay and compared between individuals responding to treatment and individuals not responding to treatment. Our results show that KYN significantly increased over time in the non-responder group, but not in the responder group. At both measurement times, 3-HAA, NA, and 3-HAA/KYN were significantly higher in non-responders compared to responders. Independently of treatment response, 3-HAA significantly increased and NA significantly decreased in both groups over time, which may thus be seen as a general treatment effect. For 3-HK/KYN, there was a significant decrease in the non-responder group, but no significant change in the responder group.

Our hypothesis, which stated that there is a significant change in KYN and KYN downstream metabolites between responders and non-responders over time was partially supported by the results. In our previous analysis, which was carried out with the current study sample (7), KYN was found to increase in non-responders over time, while the KYN/TRP ratio decreased in individuals responding to treatment. Changes in KYN were also previously displayed in meta-analyses in individuals with depression compared to healthy controls (46). However, results regarding the KYN/TRP levels differed among studies, with some studies indicating no difference in KYN/TRP levels between individuals with depression and healthy controls, or a significant treatment effect on KYN/TRP levels (46–48). Lower KYN/TRP ratios after treatment in psychiatric settings were previously associated with a decreased inflammatory drive in the KYN pathway (49). Our finding thus supports other study results, which highlight the changeability of KYN through multimodal depression treatment.

Regarding the downstream KYN downstream metabolites, studies on treatment effects are sparse to date. Our results showed significantly higher 3-HAA and 3-HAA/KYN in non-responders. 3-HAA has putatively neurotoxic properties, which were previously associated with depressed mood (50, 51). Higher 3-HAA/KYN ratios have also been associated with more severe depressive symptomatology (52).

As for NA, we found higher levels among non-responders and a significant decrease in NA over time in both groups, indicating that decreases in NA are associated with response-independent treatment effects. This finding has not yet been extensively studied before, but a study on postpartum depression found a close association between 5-HT and NA (53).

In our study, changes in 3-HK/KYN levels decreased in the non-responder, but not in the responder group. The 3-HK/KYN ratio represents the activity of the enzyme KMO activity, which reflects the metabolization of KYN to 3-HK (54, 55). Both metabolites are also able to cross the blood-brain-barrier. The KMO is known to be involved in immune function, neurobiology, and the synthesis of important signaling molecules (56). Previous findings showed that the 3-HK/KYN was elevated in individuals with affective disorders compared to mentally healthy controls (e.g., 57, 58). Our results indicate that 3-HK may play an important role in the treatment of depression. Nevertheless, for the topic of response to treatment, alternate pathways, as from KYN viaAA and further to HAA might be involved.

Apart from the significant effects, many of the investigated KYN downstream metabolites did not significantly change depending on responder status. This may be explained by various aspects. First, the short amount of treatment time might have been insufficient to elicit significant changes in many KYN downstream metabolites. For instance, Halaris et al. (59) reported that treatment with SSRIs produced a change in 3-HK, QA, KYNA/QA, QA/TRP, QA/3-HK, and 3-HK/KYN, but only after 8-12 weeks. Moreover, other factors than metabolites might have contributed to the change in BDI scores and causality cannot be given in any direction. Secondly, the lacking differentiation of medication types might have led to non-significant changes in some KYN downstream metabolites and their ratios. For instance, Myint et al. (38) observed no significant changes in biochemical parameters of the TRP pathway, although clinical symptom scores were decreased. The authors claimed that their non-significant findings might be due to the different medications used and the insufficient amount of time to reverse biochemical imbalances.

Nevertheless, the current study is one of the first to depict the effect of long-term multimodal treatments on the KYN pathways in individuals with depression. Despite the rather short amount of time, a noticeable change in these pathways was found. This highlights the changeability of KYN and various KYN downstream metabolites through the combination of several treatment methods and offers an insight into the underlying biological mechanisms of treatment response. Future studies should extend the observation period to examine the effect of multimodal interventions on the KYN pathways and the clinical symptomatology. Moreover, the results reflect the diverging literature on KYN compounds in depression, which may stem from the fact that the downstream metabolites may have both neurotoxic and neuroprotective aspects. Considering previous findings and the current results, our findings strengthen the assumption that neurotoxic and neuroprotective properties of the KYN downstream metabolites are valuable markers of treatment response in depression. Pathways from KYN in the direction of AA and further down have not been explored yet in the context of depression and treatment response, but are known to be associated with diabetes mellitus, a common comorbidity in severe mental diseases (60).

### 4.1 Limitations

The results of the study are limited by several aspects. First, this study is a naturalistic clinical study, thus medication intake and somatic comorbidities were present in many participants. Due to the inhomogeneity of psychiatric drugs and doses, the possible influence of psychotropic medication was not analysed. However, results should be interpreted considering the impact of psychopharmacological treatments on inflammation and KYN pathway activation. Moreover, since participants received several different treatments, the observed changes in KYN downstream metabolites cannot be associated with a specific therapeutic intervention. Further, participants differed in their stage of recovery. All of them had the diagnosis of a depressive disorder, but severity was different at the time of admission. It should be noted that certain symptomatic dimensions, which were not included in the study analyses, may have an impact on clinical and metabolic values and should thus be considered in future studies. As in many studies investigating KYN pathways, the amino acid concentrations were only measured in the serum and may thus not accurately reflect central concentrations. Another shortcoming is the lack of a mentally healthy control group, which would have given more insight into the underlying mechanisms of the observed effects. However, treating healthy individuals with such an intensive multimodal treatment would be unrealistic and not indicated. Finally, other variables like suidicality (e.g., 61) might play an important role in kynurenine pathway alterations and should thus be considered in future studies. Specifically, future studies should conduct sex-stratified analyses to observe potential sex-related changes in KYN pathways.

### 4.2 Conclusion

The current study investigated the effects of a six-week multimodal rehabilitation stay on the KYN pathways and their association with treatment response (responder versus non-responder). Observable changes in these pathways were found in both groups over time. These findings implicate a necessity to clarify the relevance of the KYN pathways and the related inflammatory processes in the etiopathology of depression. Specifically, their changeability through multimodal interventions, which are the common treatment option for depression, should gain more interest in research. Investigating the amount of treatment time needed to elicit changes in the biological underpinnings of depression could lead to a better clinical management and more effective treatment interventions. The association with common comorbidities as diabetes or cardiovascular diseases might help to understand the association between somatic and mental diseases and associated reduced life expectancy. Moreover, the study highlights the importance of multimodal treatment and thus promotes a more holistic understanding of psychiatric conditions.

## Data availability statement

The raw data supporting the conclusions of this article will be made available by the authors, without undue reservation.

## Ethics statement

The studies involving humans were approved by Ethical Committee of Upper Austria, Linz. The studies were conducted in accordance with the local legislation and institutional requirements. The participants provided their written informed consent to participate in this study.

## Author contributions

ER: Conceptualization, Data curation, Formal Analysis, Funding acquisition, Investigation, Methodology, Project administration, Resources, Software, Supervision, Validation, Visualization, Writing – original draft, Writing – review & editing. ML: Formal Analysis, Methodology, Resources, Validation, Visualization, Writing – original draft, Writing – review & editing, Supervision. ES: Formal Analysis, Methodology, Resources, Validation, Visualization, Writing – original draft, Writing – review & editing. FF: Conceptualization, Formal Analysis, Methodology, Project administration, Resources, Supervision, Validation, Visualization, Writing – original draft, Writing – review & editing. TS: Data curation, Formal Analysis, Methodology, Resources, Supervision, Validation, Visualization, Writing – original draft, Writing – review & editing. MS: Software, Data curation, Formal Analysis, Methodology, Resources, Supervision, Validation, Visualization, Writing – original draft, Writing – review & editing, Investigation. NM: Formal Analysis, Methodology, Resources, Supervision, Validation, Visualization, Writing – original draft, Writing – review & editing, Project administration. BR: Formal Analysis, Methodology, Project administration, Resources, Supervision, Validation, Visualization, Writing – original draft, Writing – review & editing, Conceptualization, Data curation, Funding acquisition, Investigation, Software. ND: Conceptualization, Data curation, Formal Analysis, Funding acquisition, Investigation, Methodology, Project administration, Resources, Software, Supervision, Validation, Visualization, Writing – original draft, Writing – review & editing.
